# NEGOTIATING HEALTH: patients’ and guardians’ perspective on “failed” patient-professional interactions in the context of the Swedish health care system

**DOI:** 10.1186/s12913-018-3160-4

**Published:** 2018-05-11

**Authors:** Roland Koch, Stefanie Joos, Elsa-Lena Ryding

**Affiliations:** 1 Institute for General Practice and Interprofessional Care, University Hospital of the Eberhard-Karls-University, Osianderstr. 5, 72076 Tübingen, Germany; 2 Department of Women’s and Children’s Health, Division of Obstetrics and Gynecology, Karolinska Institutet, 171 77 Stockholm, Sweden

**Keywords:** Negotiating, Professional-patient relations, Narration, Narrative analysis, Writing, Patient acceptance of health care, Writing competition

## Abstract

**Background:**

Sweden has a largely tax-funded health care system that aims at providing equal access for everyone. However, the individual’s perception and experience of the health care system remains a relevant topic for researchers. The aim of this study is to learn the patient’s perspective on how patients and professionals negotiate in the social context of the Swedish health care system.

**Methods:**

Eight essays that had spontaneously been contributed to a medical writing contest were analyzed using narrative methods. Narratives were defined as a sequence of clauses that correspond to an order of events in the narrator’s biography. The analysis comprised a three-step process. First, the essays were read and narratives were extracted. Second, an agency analysis was performed. Third, an analysis of social positioning was employed.

**Results:**

The Swedish health care system provides the social context and background for negotiations between patients and professionals. The narrators position the protagonists of the illness narratives as either patients or guardians of underage patients. The protagonists meet health care representatives in negotiation situations. Due to the lack of emotional connection between the negotiating parties, impossible situations arise. False promises are made which ultimately result in the patients’ suffering. Thus, all negotiations failed from the narrators’ perspective.

**Conclusion:**

The narrators invited their audience to solve negotiation situations differently. This study discusses some actions that may help navigate negotiation situations: Health care providers should acknowledge the patient’s or guardian’s social position and dilemma, allow emotions, involve all parties in the decision-making process and manage expectations. Writing competitions may provide a tool for experience-based assessment of health care systems.

## Background

The Swedish health care system is largely tax-financed. In 2015, Sweden’s health expenditure ranked 5th in the European region. The health expenditure share of the country’s gross domestic product was 11,1%. 83,6% of the total health expenditure was financed from public sources. Health care is culturally considered a joint effort in Sweden. Swedish health care prioritizes patient empowerment, respect for the individual and providing health care for everyone on equal terms [1].

However, personal experiences of individuals within the Swedish health care system may diverge from the established goals of the system. A continuing interest in the study of narratives within the context of Swedish health care demonstrates this: Håkansson et al. studied homeless peoples’ experiences within a shelter that provides lodging and health services. They established that the perception of illness is relative to the social circumstances in which the experience takes place [2]. In the context of sick leave and return to work, people suffering from mental illness feel stigmatized relative to those who have been absent with somatic illness [3]. Marttila et al. demonstrated that people receiving social assistance show resilience and agency, although facing multiple and complex adversities [4].

These three studies emphasize patient self-management in the social context of the Swedish health care system. Negotiations, defined as discussions aimed at reaching an agreement [5], are a recurring element in stories about illness experiences [3, 6]. According to Arthur Frank, three sub-genres of illness stories are known: Restitution, quest and chaos narratives [7].

The study of stories (narratives) and acts of storytelling (narration) generates diverse material for psychological, socio-anthropological and linguistic studies [7–12]. The storyteller (narrator) arranges past experiences into a meaningful sequence. This sequence is then presented to an audience. Thus, narration is both communication and social interaction [13, 14]. Often, the narrator creates a self-image (narrative identity) through which she re-lives and re-enacts past experiences. Characters appearing in the narrative are positioned by the narrator to negotiate social positions [15]. Narratives act as meaning-making devices, allowing the narrator to make sense of past events and realign them to her personal biography and self-concept [16].

Narration is inseparably interwoven with the narrators’ social reality and cultural background. The analysis of stories (narrative analysis) is thus suited to research personal experience in the sociocultural context. In summary, narratives are complex and intersubjective constructs of experience. They are used by narrators to negotiate social positions [17]. The WHO has acknowledged that narration is important to understand cultural aspects of health and health policy making [18].

The aim of this study is to learn the patient’s perspective on how patients and health care providers negotiate differences in the social context of the Swedish health care system.

## Methods

### Recruitment

The source of our study material are contributions to the 2016 medical writing contest (“skrivaretävling” [19]). This contest is hosted biannually by the Swedish Balint society (SFMP). The competition usually invites medical professionals (nurses, doctors, social workers) to write a self-reflective essay on a personal patient-health care provider experience.

In the 2016 competition, however, the jury found nine contributions that were written from a patient’s perspective. A brief request revealed that the authors were indeed patients. They had found the competition advert on the internet. Since this was unexpected and provided a unique occasion to study spontaneous narratives about patients’ perspectives, one of the jury members (ELR) decided to initiate this exploratory study. Inclusion and exclusion criteria as applied to the material are shown in Table 1.

**Table 1:** Inclusion and exclusion criteria

Inclusion criteria	• Essay author identified herself as a patient • Narrative was written from a patient and/or guardian perspective • Written consent
Exclusion criteria	• Essay author identified herself as a medical professional • Concerns about safety or discomfort • Request of legal release clause • Request of payment

We contacted the nine authors and explained the study goal and ethical principles for voluntary participation. Eight of the nine authors gave their written consent to participate. Thus, eight essays were included in the study. The essay authors were then asked to provide information about their gender, age and profession.

To ensure participant safety and to enrich the credibility of our findings, a member check was planned. A member check is a qualitative research technique in which a summary of the findings is presented to the participants. Participants’ reactions and feedback to the results is then obtained. None of the eight participants withdrew their consent after the member check. Their feedback is included in the discussion section.

All participants were middle-aged, working Swedish women. Their professions were stated as: Airport control officer, author, business consultant, police officer, teacher, teaching assistant, university professor. One participant did not provide information about her profession.

### Analysis

Narrative Analysis reconstructs the inner logic of stories in order to analyze how individuals build and experience their social reality. It examines three basic questions [11, 20]:

1. What is being told?
2. How is it told?
3. Why is it told in *that* way?

While the first analysis step requires a dedicated reader with an open mind, the two subsequent steps ask for a more empirical and structured approach. Following the three basic questions, the analysis process lasted from 01/2016 to 08/2017. RK had most experience in this analysis method and thus performed all three analysis steps. Between each step, a two-three month pause was planned.

#### Step 1: Getting to know the texts and finding narratives

First, two authors (RK and ELR) read printouts of all essays alone. During reading, spontaneous thoughts and feelings were noted by hand. The two authors then discussed their thoughts and findings. During this discussion, possible topics emerged. RK characterized the stories according to Frank’s definitions of illness narratives. RK and ELR then extracted narratives from the essays based on Labov’s definition of a narrative. According to Labov’s linguistic definition, a narrative is a sequence of clauses that correspond to an order of events in the narrator’s biography [8]. Episodic narratives were collected into “vignettes” [9]. This provided a framework for the following analysis steps.

Step 1 thus helped getting acquainted with the material and the narrators. It allowed us to approach the question: “What is being told?”

#### Step 2: Agency analysis

Second, RK analyzed narrative agency. Basically, a narrator directs the characters of her story. She uses stylistic elements such as dramaturgy, choice of genre and language to attribute (or deny) power to her characters. By examining the degree to which characters in a narrative are able to influence their lives or the lives of other characters, the narrator’s view of her own impact on the fate of her characters become clear. This allows researchers to understand the narrator’s concepts of control, responsibility and autonomy [15].

Methodologically, step 2 focused more on the second question: “How is it told?” All three authors discussed the results of this analysis step. Several topics and points of interests arose that needed further explaining.

#### Step 3: Analysis of social positioning

Third, RK performed a positioning analysis. Narration is a real-time act of constructing and negotiating social positions between narrator and audience through the attribution of features, characteristics, intentions and roles. The narrator actively positions her characters towards each other. She also positions herself to her story and towards the audience. Positioning analysis thus offers insights into the narrator’s social stance and her sociocultural background [21].

In this analysis step we could integrate results from the first two analysis steps into the social context of the writing competition. In short, the results of the final analysis step answered the question “Why is it told in *that* way?”. The results were discussed in the study group and later in an interdisciplinary qualitative research workshop group that consisted of social and medical scientists.

For the sake of readability, only the final results (step 3) are presented.

## Results

All of the following text examples have been translated from Swedish. Replacements, insertions or clarifications use square brackets. Table 2: Overview of the narratives contains information about the heterogeneity within the narratives in greater detail.

**Table 2:** Overview of the narratives

Protagonist	Patient or Guardian to Patient	Antagonist	Word count	Time frame	Acute or chronic disease	Inpatient or Outpatient setting
Middle-aged Woman	Guardian to a young girl	Several doctors	956	Several weeks	Acute	Both
Middle-aged Man	Guardian to an adolescent girl	Several doctors	1276	Several weeks	Acute	Both
Middle-aged Woman	Guardian to an adolescent girl	Several doctors & psychotherapist	1143	Several weeks	Chronic	Outpatient
Middle-aged Women (Group)	Patient	Saleswomen in hospital context	951	2–3 Hours	Chronic	Inpatient
Middle-aged Woman	Patient	Oncology Nurse	922	Less than one hour	Chronic	Outpatient
Adult man	Patient	One Doctor	553	Less than one hour	Unknown	Outpatient
Adult, no gender specified	Patient	Nurse, other Patients	1704	Several weeks	Chronic	Inpatient
Adult, no gender specified	Patient	Several Doctors	302	Several weeks	Chronic	Both

It became clear during the first readings that seven narratives were about negative experiences of patient–health care provider encounters. Following Frank’s classification of illness narratives, the narratives were identified as quest narratives. In quest narratives, the protagonist confronts a series of challenges. The goal of the quest is to preserve or to gain health. In the study material, these challenges were presented as negotiations between health care providers and the protagonists.

The narrators chose tragedy as their narratives’ genre: The narrators slowly built up conflicts and tension in the negotiation situation. The tension ultimately resolved in a climax, which led to a negative ending. This ending often meant a negative impact on patients’ health and well-being. Narrators staged conflict between characters. They used emotional language with poetry-like verses and metaphors to create an emotional undertone in the narratives. The following first excerpt shows the closing paragraph of a narrative. The story was about an adolescent girl with a presumed neuropsychiatric condition. She was accompanied by her parents, mainly her mother, during several appointments with child psychiatry staff. The story was told from the perspective of the mother.

A’s father and I walk the corridors of the Child Psychiatry Clinic on either side of A; alone; finely crushed glass in our veins. Intolerable pain for every drop of blood fighting its way through the heart. (S25)

Four major categories emerged that contributed to this negative outcome in patient-health care provider negotiations:

### Disrupted patient-health care provider relationship

The protagonists scrutinized health care providers’ words and actions. This created a tense atmosphere in which the care provider’s best intentions risked being disregarded or misinterpreted. The following example shows one narrator’s interpretation of basic hygiene rules. She used the example to illustrate the emotional detachment of health care providers. She positioned herself as a caring mother who is the expert on her child:

Does [the doctor] see the child at all? Or is she just afraid to become infected? She touches the child with rubber gloves. She is so sure of the diagnosis: A common gastroenteritis. Why does that woman not listen to a mother’s worries? Does she not know what it is like to be awake at night? (S14).

The last question especially challenged the moral and emotional position of the physician. In the mother’s eyes, the physician did not understand the difficulties of tending a sick child. A conflict of roles was also present: The narrator expected that a female doctor should understand her as a woman.

The interpersonal positions and relationships were especially complex in the narratives that involved parents, children and health care providers. Since the health care providers invested a lot of energy in the patient, the guardian character often felt disregarded.

Guardians were conflicted: On the one hand, they depended on the health care provider’s expertise and did not want to disturb the diagnostic process. On the other, they wanted their own needs to be fulfilled. For example, they wanted to be involved as experts on their children. This conflict might have contributed to the protagonist’s increasing frustration, which was reflected in the dramatization of the narratives.

In the following example, the protagonist’s daughter, who was the patient in this narrative, felt that a doctor’s apology was acceptable. However, the protagonist himself, the father, was dissatisfied and remained unforgiving towards the physician. Forgive physician. Forgiveness had to be provided by the daughter:

“I want to offer you an apology”[, the doctor said]. N sees B [his daughter] nod. He bites his tongue. He does not want to tell the doctor about the missed delivery at work and how his boss treated him because of that. He forces himself to say:

“So what do you think, B?”

“It’s all right, dad”, she says, “it’s all right”.

Well, it had better be. (S17).

Care providers were perceived as representatives of “the system”, not as individuals. However, because the health care providers were accessible during the negotiations, the protagonists’ (and narrators’) frustration was mainly directed at them. The stories contained generalizations of negative experiences, as the following excerpt illustrates:

Two small words [Title of narrative].

This is how Swedish doctors have marked me.

“Unwashed, sober.”

This was written in my medical record nearly ten years ago. (S23).

The description of the patient as “unwashed, sober” was made by a single physician in the medical record. The narrator, who in this case identified with the protagonist by using a first-person narration, however, generalized this as “Swedish doctors” (plural) having marked her. This demonstrates that one single failed patient-health care provider interaction may change a patient’s position towards the health care system in general.

### Difference in social position

The protagonists were characterised as people with low self-esteem. Negative experiences within the health care system were associated with negative consequences for the protagonists. Past trauma (both physical and psychological) still influenced protagonist behaviour and attitudes as reflected in the narrative. This is a recurring topic in the narratives:

He could not win arguments; Not against doctors. If he could, he would not have to walk around with his bad leg. He would at least have obtained an examination at that time (S17).

The protagonists remained at a disadvantage during the negotiations. In contrast, the health care providers navigated “the system” freely. Health care providers’ advantage in knowledge, legal power and the support of a larger group led to a better social position. From the patients’ perspective, providers granted or declined health care services, as the following example illustrates:

A[Mother]:: “Okay, I’m coming right now.”

B[Nurse on the phone]: “We can offer you an appointment at eight.”

A: “Now!”

B: “Eight o’ clock.”

A: “I’m coming now! Let me just put some clothes on the kids”.

B: “But you will have to sit in the waiting room for several hours till it’s eight!” (S36).

In another example, the guardian assisted her child in communicating with a psychiatrist and a psychologist. The stigma involved with being mentally ill or being the parent of such a child was tangible as an awkward feeling of shame. Patient and parent were excluded from the group of health care providers. The health care providers remained “professional” by refraining from any emotional response:

“Our test results show that there are major difficulties, much more pronounced than in the preceding tests” [the psychiatrist says].

A [The daughter] interrupts:” No cows inside, moo! “and laughs heartily.

Her father and I join in the laughter.

The psychiatrist looks at me questioningly: “What did she say?”

“It’s a joke. A has difficulties saying ‘shoes‘, she says ‘cows’. So we tell her that cows say ‘moo’, it’s called ‘shoes’”. [“Shoe” and “Cow” sound similar in Swedish].

The psychiatrist and the psychologist look puzzled. They do not smile.

“Yes, as I was saying” [the psychiatrist continues] (S25).

The feeling of shame arose when the health care providers did not laugh with the family. The laughter would have relieved the awkward tension. Instead, shame and anger arose. The topic of stigmatization remained hidden. This contributed to the negative spiral of anger, shame and frustration which ultimately led to a failure of the negotiation.

### Circumstances influencing negotiations

At times, the course of negotiations was influenced by circumstances beyond the control of the protagonist or the health care system representative. In the following instance, a single mother was speaking on the phone with an oncology nurse. Parallel to this very serious phone call, she had to take care of her two children that were fighting in the adjacent room. This excerpt also serves as an example on how appointments are handled in the Swedish health care system: Generally, patients call an automated phone centre, type in their phone number and later receive a call-back appointment. That return call might occur at a bad time:

A [Mother]: “But why this evening? Why the rush?”

Directed at the adjacent room, she bellows: “Be quiet now, I’m having a phone call!! And it’s important!!!”

B [Nurse on the phone]: “We had a free appointment there. Furthermore, we quickly need to see if it’s just picture artefacts or... something else.” (S36).

The nurse was keen on delivering the best care possible. She had the best intentions. The narrator positioned the mother in a situation where she had a disadvantage. Taking care of her children left no time for reflection. The possibility of a serious illness caused her an enormous amount of stress and fear. This is an example of how narrators opposed medical care to the emotional and social needs of the individual.

In another narrative, there was no negotiation at all because the doctors did not wait for the father’s consent to decide upon the discharge of a seventeen-year old girl from the surgical ward:

But the doctor had already been there, had decided. And there were others that needed the bed. (S17).

The last passage illustrated that the needs of many were more important than the need of the individual. The doctor’s argument “There were others that needed the bed” was accepted as a fact. However, in the continuation of the story, the premature discharge led to severe complications for the patient. Following the arguments of the narrator, there are situations where the needs of many lead to severe suffering for the few. This story’s narrator clearly rejected the notion.

### Unrealistic expectations

Narrators used health care providers’ promises in a satirical fashion: All negotiations ended with a negative outcome. To make a promise in that context had to seem cynical. Below are some examples of narrators’ use of expectations and promises.

Woman: “Everyone, I understand you have breast cancer?”

Some of the women nod embarrassedly.

“All of you will feel so much better when you see how much healthier you look after having used these products”. (S26).

The narrator of the above example opposed breast cancer to the use of beauty products. The text was entitled “look good, feel better”. This positioning was a clear statement against a superficial and emotionally detached health care system.

She [the mother] sees the ribs sticking out on the child.

That physician understands: You can go crazy when the stomach flu never gets better!

He had small kids once, too. But on fluid replacement every tenth minute even cholera patients survive.

She feeds the little girl with a spoon every tenth minute, but it does not get better. […] She cannot stand it any more! (S14).

This doctor’s use of communication techniques and empathy had no influence on the position of the narrator. The mother did as she was told, but the promised positive result did not occur. Instead, the child’s condition worsened. The mother’s disappointment added to her frustration, which in turn was expressed in the dramatization of the overall narrative.

### Negative case analysis

One story was considered - although brilliantly written - a negative case because of its clearly fictional character. The story was about a patient in a closed psychiatry ward. There were no negotiations in the story. The hero had no specified gender, but we assumed he was male. He had a pocket watch through which he could manipulate time.

Being a great listener, he knew almost everything about the staff members and the other patients. He acted as a counsellor to other patients and staff. The story ended when he decided to die. He then used the clock to speed up time. Before that, he wrote a to-do-list to his nurse:

I write some lines to the nurse:

1. Leave your disgusting husband.

2. Get a different job.

3. I know you have little time. Thus, I want you to have my pocket watch. It is of utmost importance that you turn the upper two dials one click in the direction of the sun before wearing it. (S28).

The decision was always the patient’s. The story had a high degree of agency because the patient remained the master of his own fate at all times (even when forcibly restrained to the bed). Thus, the story provided the antithesis to the other narratives that were based on personal experience. It represented a utopia about the patient being in control. Everybody else, including medical staff, was positioned as a prisoner of the system. Changing jobs or dying were the only ways to escape this situation.

## Discussion

The findings provided new insights into failed interactions between health care providers and health care seekers in the Swedish health care system. Negotiation was the main topic of the interactions. In the narrated situations, the protagonists faced nearly impossible odds. Most negotiations failed from the narrators’ perspective. As a consequence, the protagonists and/or their protégés suffered. With the exception of the above-mentioned negative case, these quest narratives only featured tragic heroes.

The writing competition was intended for health care providers. Regardless, arguably privileged middle-aged Swedish women decided to send their contributions. Returning to the basic questions of narrative analysis, one asks: Why tell that specific story in that way in that specific context?

### Why do patients contribute to a medical writing contest?

Tavernier et al. demonstrated that young adults that adolescents whose narratives included meaning-making in regard to critical life events scored better at well-being tests. Meaning-making in stories is thus correlated with well-being [22]. Little or no meaning-making or self-reflection could be seen in the stories of this study. Narratives from objectively more exposed groups of Swedish people [2, 3] contained clearer testimonies of agency and self-management than the stories analysed here.

Our participants might have written the essays with the intent to criticise the health care system. All authors - working middle-class Swedish women - had full access to health care and social benefits. Their main criticism was the lack of emotional response in negotiation situations.

This criticism was expressed by the extensive use of negative emotional language in the narratives. We demonstrated that patient-health care provider interactions sometimes become negotiations. Negative emotions can have a negative impact on negotiations [23].

Illness is associated with stigma. In the narratives, stigma was strongly associated with shame. Our findings demonstrated that shame can be experienced by patients and by parents or guardians of sick children. Despite the health care providers’ best effort to act “professionally”, the parents in the narratives felt excluded from patient-care provider relationship. This in turn contributed to parents’ feelings of helplessness and frustration. It also increased patients’ and parents’ dependency on the health care system. In their study on the stigma of substance use, Chang et al. reflected [24]:

According to Bourdieu, habitus comes from individual experiences, but since individual experiences are structured by our social position, one cannot separate habitus from an individual’s structured experiences as a member of various social groups. That is, our everyday actions today are shaped, though not determined, by our structured, stratified pasts. And in the field of health care, where differences and inequalities are worked out in clinical interactions underpinned by specific norms and rules, substance use stigma is produced, exacerbated and negotiated (page 18).

The narrators chose to end their narratives with the protagonist’s negative feelings. These feelings arose as a result of the failed negotiations. The protagonists’ future remained unclear to the audience. Implicitly, the audience (i.e. the writing competition jury, an audience of presumed health care providers) were prompted to find a better way. The open ending of each story allows different ways to read the story. This is called “subjunctivity” [17]. Wanting to change that outcome motivated us to conduct this study in the first place. It was a typical “doctor’s reflex” to a patient’s plea.

In summary, the patients saw the writing competition as a means to publish their stories. They knew that their stories would reach health care representatives who were interested in the emotional aspects of patient-health care provider interaction.

### Why did the contributors choose negotiations as a topic?

The narrators focused on negative health care experiences and the impact of those experiences on the individual. Since the narrators identified with the protagonists, patients or their guardians were cast as heroes. This automatically put the health care representative in the role of the antagonist.

A narrative researcher always becomes part of narration because he or she is a recipient of the narrative [20]. This is especially true for illness narratives [25]. Therefore, we compared our findings with other studies on negotiations in health care.

Stoddart observed meetings between community nurses and patients. She then held semi-structured interviews with both negotiation parties and asked them about the relationship [26]. The material was qualitatively analysed with grounded theory. She identified three core aspects of negotiation: Navigation, socio-cultural characteristics and power and control. She came to similar conclusions: Observed negotiation situations and narratives of such situations share common elements. Narratives and narrative analysis, however, emphasize subjectivity. If personal experience and social position are the main point of interest, narrative analysis seems to be the most fitting research method.

Nilsen studied doctors’ negotiation strategies in assessing sick leave in patients. He first held focus groups and then analysed the material with systematic text condensation [27]. Participating physicians expressed the need to balance biomedical issues and their responsibility towards society. They felt that they were “street-level bureaucrats”. The findings of our study show the perspective of the health care seekers. The narrators felt no special responsibility towards society. On the contrary, a repeating and seemingly futile struggle to “get what they needed” is a recurring topic of the narratives. The needs of the individual were repeatedly opposed to the needs of many. This contrast is a challenge for health care providers, who need to balance the patient’s emotions and individual social position with society’s needs. Indeed, one GP in Nilsen’s study reflected (Page 42):

“When I deal with long-term sick leave for conditions I don’t quite understand, I always talk to the patient about his work, his marriage, his children and his financial situation. A lot of trouble lies hidden here”.

Mustafa examined general practitioners’ (GP) use of negotiation strategies when they met patients that expected antibiotic treatment [28]. The study was based on thematic analysis of semi-structured interviews. GPs reported that if they learned and managed patients’ expectations, negotiations had a more positive outcome.

In another study that used a quantitative approach, Raieli studied parents’, children’s and doctors’ expectations in situations where the treatment of the child’s headache was indicated [29]. Patients’ or guardians’ expectations differed from those of the paediatrician. For example, paediatricians expected that fear of a brain tumour was more important to patients and parents than the acute management of pain. The opposite was the case. Both studies conclude that patient expectations must be obtained and managed.

Our study showed that unclear expectations or false promises disappoint patients. The negative experience resulted in the loss of trust in the health care system. Trust modulated patient behaviour, as other studies have shown [30]. If a patient had too little trust in health care, he or she withheld vital information or postponed seeking help even though symptoms had worsened. Patients were also at risk of generalizing disappointing experiences. A single negative experience that perhaps was derived from a failed relationship with a health care provider shaped their view on the health care system as a whole. To manage expectations is a way to manage patients’ trust in the health care system.

Negotiations provided the narrators with a stage. There, the narrators directed complex dramatic plays that featured conflicting emotions and social roles. In all narratives, the negotiations’ negative outcome was used as the central dramatic element.

In summary, negotiation situations were a fitting stage for the narrators’ point. Addressed at the audience of health care providers, the narrators declared: “There is something wrong with the system – do something”.

### Strengths and limitations

Due to analysing eight narratives from a highly selective source (a medical writing competition), the scope of this exploratory study in terms of sample size and depth is somewhat limited. The “interview question” basically was the advert of the writing competition [19]: “the essay should reflect ethical and cultural aspects of patient-care provider relationships in clinical encounters”. This frame allowed enough freedom to tell a story [7]. In contrast to a more conclusive research approach, several questions remained open. At the same time, we established that writing competitions are a viable data source in health care research.

All study participants were middle-class working Swedish women. Thus, less privileged groups and men were not represented. On the other hand, we discovered a complex interplay of roles and emotions in the context of the Swedish health care system. Our approach biased the sample towards persons with above average communication skills. Much reflection on patient-health care provider relationships was done implicitly by the use of language and characters. Thus, the selection of persons with the skills to articulate their experiences benefitted the purposes of this study. For less articulate persons, interview studies seem to provide better study opportunities, because the interviewer can engage in a dialogical narration of the story [2, 7, 20].

The material was derived from spontaneous contributions. This provided an honest insight into what Swedish women with negative health care experiences spontaneously considered important to tell an audience of health care providers. The material is diverse enough to research interactions in different settings (in-patient, out-patient) and different types of disease, like chronic and acute illness.

Even under the limitations of a somewhat biased and limited sample, the findings were credible. This aspect is very important in narrative research according to the quality recommendations on narrative research by Greenhalgh [31] and Loh [32].

The triangulation of analysis methods in the three-step analysis process helped maintain a breadth of perspective: Agency analysis identified that the narrated interactions were indeed negotiations. After integrating this aspect in the analysis, several narratives could be understood much better. Positioning analysis helped discover the health care system critique. This allowed the analysis of another narrative, which at first was regarded as a negative case. Furthermore, study material and results were presented to experts (sociologists and fellow GPs) in a qualitative methods workshop. Their feedback confirmed that we were on the right track.

During member check, we asked the participants to provide feedback about our findings. They recognized the problem of different positions in power. When phone calls were involved, they added that this power is often misused to keep patients away from vital doctor time. Unnecessary waiting for the doctor (or the absence of doctors in general) was seen as a consequence of how the Swedish health care system works today. Furthermore, they agreed that patients and guardians wanted to be recognized as experts on their own body (or the body of their protégé). Their feedback puts the above-mentioned sample bias into perspective: Even though the narrators were arguably privileged, literate and well-spoken, power-play occurred. The resulting negative emotions sabotaged the outcome of negotiations. Thus, the member check added to the credibility of our findings.

Lastly, our own background as doctors heavily influenced the interpretation of the findings. Reading the patient’s narratives triggered countless experiences with patients. Many stories that dwelled within us resonated with the analysed narratives. On the one hand, this limited our objectiveness. On the other, it contributed to our findings’ credibility and possible application in practice. We believe that social scientists and physicians should cooperate on medical narrative research topics and look forward to an exchange about that.

## Conclusions

All analysed stories challenged the audience to find a more patient-centred approach in dealing with negotiation situations. We conclude that a disrupted patient-health care provider relationship, difference in social position and mismanaged expectations contribute to the failure of patient-health care provider negotiations. In turn, a better management of expectations and the acknowledgement of emotional and social aspects of patient-health care provider interaction may provide a key to improve patient experience.

A writing competition invites patients or their guardians to share their experiences. We would like to continue this line of research and implement writing competitions as a tool to obtain tacit knowledge of health care system users. Based on our experiences with this exploratory study, we would like to design a more conclusive study, which focuses on some of the open questions of our findings. A comparison of patient experience in different health care systems seems especially tempting.

Personal experience and patient participation should play a larger role in the quality assessment of health care systems and in the research of patient-health care provider communication.

## Abbreviations

(Sxx): Writing Contest Submission number xx (Reference to narrative)
GP: General Practitioner
SFMP: Svensk förening för medicinsk psykologi (Swedish Balint society)
